# Exploring retinal ganglion cells encoding to multi-modal stimulation using 3D microelectrodes arrays

**DOI:** 10.3389/fbioe.2023.1245082

**Published:** 2023-08-01

**Authors:** Kui Zhang, Yaoyao Liu, Yilin Song, Shihong Xu, Yan Yang, Longhui Jiang, Shutong Sun, Jinping Luo, Yirong Wu, Xinxia Cai

**Affiliations:** ^1^ State Key Laboratory of Transducer Technology, Aerospace Information Research Institute, Chinese Academy of Sciences, Beijing, China; ^2^ School of Electronic, Electrical and Communication Engineering, University of Chinese Academy of Sciences, Beijing, China

**Keywords:** 3D microelectrodes arrays, retinal ganglion cells, multi-modal stimulation, neural encoding, electroplating

## Abstract

Microelectrode arrays (MEA) are extensively utilized in encoding studies of retinal ganglion cells (RGCs) due to their capacity for simultaneous recording of neural activity across multiple channels. However, conventional planar MEAs face limitations in studying RGCs due to poor coupling between electrodes and RGCs, resulting in low signal-to-noise ratio (SNR) and limited recording sensitivity. To overcome these challenges, we employed photolithography, electroplating, and other processes to fabricate a 3D MEA based on the planar MEA platform. The 3D MEA exhibited several improvements compared to planar MEA, including lower impedance (8.73 ± 1.66 kΩ) and phase delay (−15.11° ± 1.27°), as well as higher charge storage capacity (CSC = 10.16 ± 0.81 mC/cm^2^), cathodic charge storage capacity (CSCc = 7.10 ± 0.55 mC/cm^2^), and SNR (SNR = 8.91 ± 0.57). Leveraging the advanced 3D MEA, we investigated the encoding characteristics of RGCs under multi-modal stimulation. Optical, electrical, and chemical stimulation were applied as sensory inputs, and distinct response patterns and response times of RGCs were detected, as well as variations in rate encoding and temporal encoding. Specifically, electrical stimulation elicited more effective RGC firing, while optical stimulation enhanced RGC synchrony. These findings hold promise for advancing the field of neural encoding.

## 1 Introduction

Understanding how populations of neurons encode external stimuli is crucial for unraveling the complex mechanisms underlying information processing in the nervous system (Schneidman et al., 2011; Ng et al., 2020). Deciphering the encoding principles of neurons relies on identifying the information contained within spike sequences and how stimulus-related information is represented by these spikes. Within the visual system, retinal ganglion cells (RGCs) play a vital role as the final output neurons responsible for transmitting sensory information from the external environment (Masland, 2001; Trenholm et al., 2013). Investigating how RGCs encode distinct information from external inputs will enhance our understanding of the encoding mechanisms employed by the nervous system.

Microelectrode arrays (MEA) offer a high spatiotemporal resolution for simultaneous electrophysiological recordings of multiple RGCs (Marblestone et al., 2013). This versatile tool has demonstrated significant advancements in various areas, including visual encoding (Fiscella et al., 2015; Pamplona et al., 2022) and visual prosthetics (Corna et al., 2021; Greco et al., 2021). However, when applied to *ex vivo* retinal tissue, conventional planar MEAs face challenges due to the curved structure of the hemispherical retinal tissue, which can result in limited spatial resolution and potential hypoxia for neurons located at the bottom of the electrode. Additionally, the limited contact between planar electrodes and the tissue leads to reduced coupling, ultimately diminishing the signal-to-noise ratio and recording sensitivity (Reinhard et al., 2014; Fujii et al., 2016; Ha et al., 2020). These factors collectively impact the physiological measurements of RGCs, thereby hindering our understanding of neuronal encoding.

The utilization of three-dimensional microelectrode arrays (3D MEA) presents a promising solution to overcome the limitations associated with planar MEA-based detection of RGCs (Ha et al., 2020; Seo et al., 2020). The protruding three-dimensional structure of 3D MEA not only enhances the coupling efficiency with retinal tissue but also provides additional spatial capacity to effectively mitigate tissue hypoxia, which are crucial considerations for the cultivation and assessment of retinal tissue (Wang et al., 2010; Seo et al., 2019). There are various methods available for the fabrication of three-dimensional electrodes, including electroplating, etching, 3D printing, and others (Hales et al., 2020; Choi et al., 2021), Among these methods, electroplating on planar electrodes offers a cost-effective and highly customizable approach for the formation of three-dimensional electrodes (Hai et al., 2010; Teixeira et al., 2021). Researchers can selectively electroplate desired locations, choose appropriate materials, and control the height of the electrodes based on their specific detection requirements (Spanu et al., 2020). In our study, this approach will be employed to monitor the electrophysiological activity of retinas with unique structures.

Based on a comparative analysis of the performance of commonly used biosensor materials, PtNPs were selected as the choice for constructing three-dimensional electrode structures (Moon et al., 2018; Chang et al., 2019). This decision was primarily driven by their mechanical properties and stability, as substantiated through practical experiments conducted on retinal testing. The exceptional mechanical characteristics of PtNPs ensure the preservation of the electrode surface structure during the delicate process of retinal positioning, this is the key to detecting the neural activity of RGCs. Moreover, to validate their electrochemical capabilities, comprehensive tests were performed to evaluate the electrochemical performance of the three-dimensional electrodes constructed with PtNPs in comparison to planar electrodes. The results unequivocally confirmed the superior electrochemical performance achieved by the 3D MEA formed with PtNPs.

Using the fabricated 3D MEA, a study was conducted on the encoding of RGCs under multi-modal stimulation. Previous research has indicated that neurons can encode information through rate encoding and temporal encoding (Shadlen and Movshon, 1999; Uhlhaas et al., 2009; Saal et al., 2016). Rate encoding refers to the transmission of neuronal information through firing rates (number of spikes) (Mease et al., 2017). Temporal encoding suggests that the precise timing of each spike carries information (Mackevicius et al., 2012), such as the onset timing of a spike (Denning and Reinagel, 2005; Lesica et al., 2006), interspike intervals (ISI) (Butts et al., 2010), and the duration of a firing (Marsat and Pollack, 2010). Additionally, studies have shown that burst (Gollisch and Meister, 2008; Zeldenrust et al., 2018), the intraburst ISI (Oswald et al., 2007), and synchronous firing across multiple neurons also encode information (Toutounji and Pipa, 2014; Torre et al., 2016). Therefore, in this study, we conducted an analysis of Rate encoding and temporal encoding in the retina under multi-modal stimulation conditions.

Optical Stimulation (OS), Electrical Stimulation (ES), and Chemical Stimulation (CS) were employed as sensory stimuli. An analysis of the response patterns and response times of Retinal Ganglion Cells (RGCs) was conducted under different stimulation conditions, resulting in the identification of distinct responses observed across the four conditions. Furthermore, variations in spike firing rates as a measure of rate encoding in RGCs were investigated among the different stimulation conditions. Temporal encoding was explored by examining joint ISI distribution, correlation, and burst count across the four conditions. Through these comprehensive studies, valuable insights into the field of neural encoding research are anticipated to be provided.

## 2 Materials and methods

### 2.1 Reagents and apparatus

The retinal culture medium consisted of the following components: 120.0 mM NaCl, 5.0 mM KCl, 2.0 mM CaCl_2_, 1.0 mM MgCl_2_, 30.0 mM NaHCO_3_, 15.0 mM Glucose, 0.2 mM L-Glutamate, 10 ppm Phenol red, pH 7.5. The CS reagents utilized were 20 μM KCl. Fine ophthalmic scissors, forceps, and bent-nose pliers (RWD Life Science, China) were utilized as surgical instruments. The experimental animals involved in the study were C57BL/6J mice aged between 3 and 6 weeks, obtained from Charles River Laboratory Animal Technology Co., Ltd.

The following equipment was utilized in this study: a Cerebus multichannel neural signal acquisition system (Blackrock Microsystems, United States), a dual-channel flow-type perfusion pump (Lead-2, China), an ultrapure water system (MW-D20, China), an intelligent digital temperature controller (XMTD-6000, China), a pressure chamber (SHD-42/10, United States), an inverted microscope (TE2000-U, Nikon, Japan), an electrochemical workstation (Gamry Reference 600, United States), an ultrasonic cleaning machine (SK2200HP, China), LED light source, controller, and fiber optics (Thorlabs, United States), and a dual-channel electrophysiological electrical stimulator (MultiChannel, United States).

### 2.2 Retinal detachment and culture

All animal experiments performed in this study adhered to the guidelines and regulations established by the Beijing Association on Laboratory Animal Care and were approved by the Institutional Animal Care and Use Committee at Aerospace Information Research Institute, Chinese Academy of Science. Mice within the specified age range were selected to ensure consistency in visual characteristics across the experimental groups.

**FIGURE 1:**
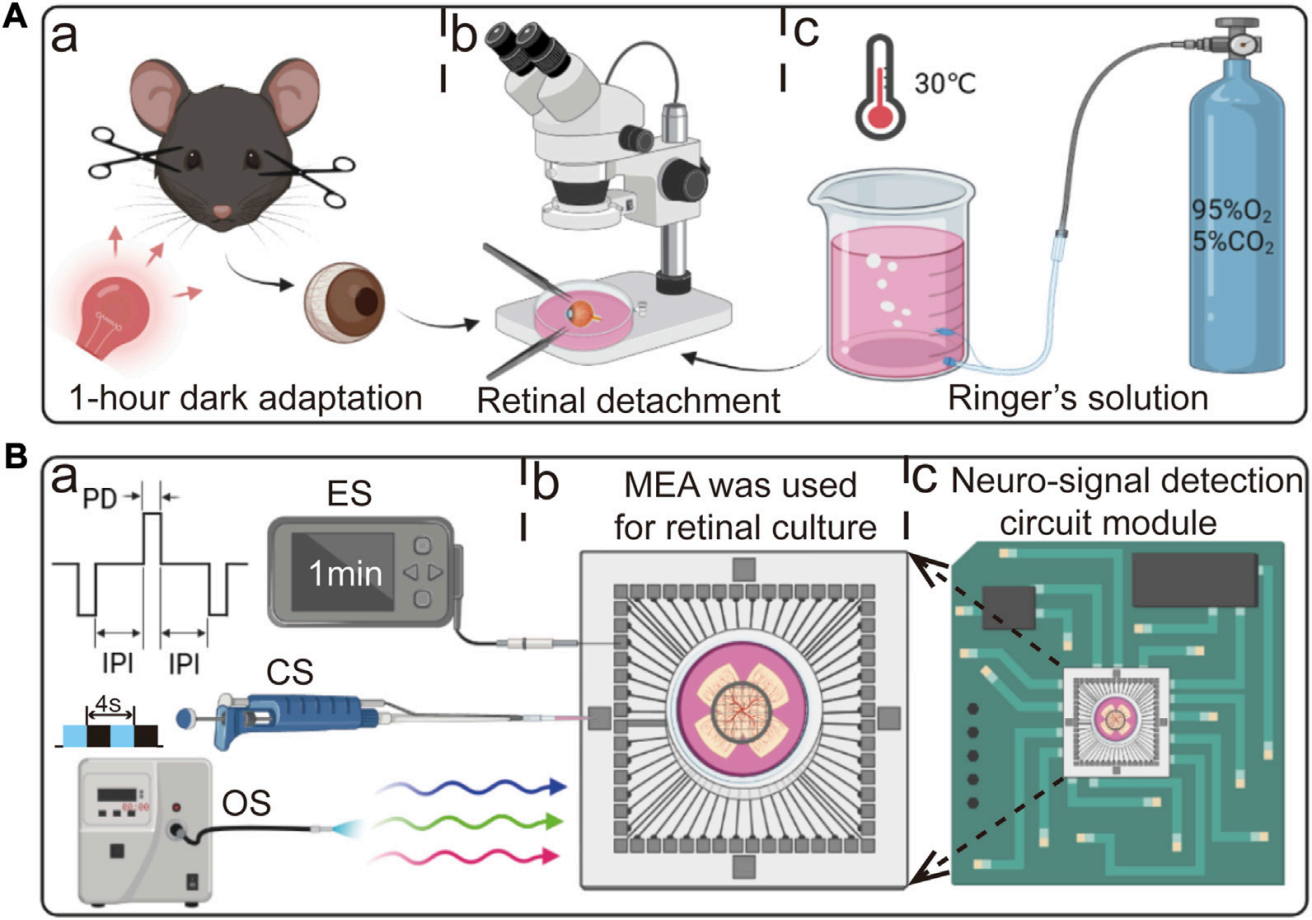
Schematic diagram of retinal detachment, experimental procedure, and detection circuit. **(A)** Experimental workflow for retinal detachment in mice. (a) After 1 h of dark adaptation, the mice were euthanized by swiftly performing cervical dislocation under the illumination of dim red light. (b) Retinal detachment in retinal culture medium. (c) Retinal culture medium supplemented with 95% O_2_ and 5% CO_2_. **(B)** Three stimulation protocols and detection circuit used in this study. (a) From top to bottom: electrical stimulation (ES, PD: pulse duration, IPI: inter-pulse interval), chemical stimulation (CS), and optical stimulation (OS). (b) Retina adhered to the surface of the MEA. (c) MEA inserted into the detection circuit.

As depicted in Figure 1A, a dark adaptation period of 1 h was provided to the mice in a dark environment. Concurrently, the retinal culture medium was infused with a gas mixture of 95% oxygen and 5% carbon dioxide at a temperature of 30°C for 30 min. After completing the preparatory steps, the mice were euthanized by swiftly performing cervical dislocation under the illumination of dim red light. Subsequently, their eyeballs were delicately extracted and transferred to pre-prepared dissecting dishes. Using fine ophthalmic scissors and forceps, the eyeballs were halved along the serrated edge, and the cornea, lens, and vitreous body were subsequently removed. The retina was delicately separated using forceps, transferred onto an MEA, and inserted into the detection circuit.

To ensure the experimental success rate, the activity, and adhesion of the retinal samples were evaluated, as illustrated in Supplementary Figure S1. The selected retinal samples were subjected to perfusion at a flow rate of 1 mL/min, and the initiation of perfusion was recorded as t_0_. After 10 min from t_0_, the activity of the retina was assessed. If the test result indicated sufficient activity, signifying a positive outcome, the experiment proceeded according to the predetermined experimental plan. The entire experiment had a duration of approximately 1 hour.

### 2.3 Methods of sensory stimulation

Upon successful detection of the response from RGCs, a 5-min baseline recording of spontaneous firing activity was obtained as the control group. Subsequently, the influence of different stimulation methods on the population response of RGCs was investigated. The sequential order of multi-modal stimulation was as follows: OS was administered first, followed by ES, and finally CS. Immediately after each stimulation, electrophysiological signals were recorded for 5 min. A 5-min rest interval was incorporated after each recording period before proceeding to the next stimulation group.

As shown in Figure 1B, for OS, a flash stimulation method was employed to stimulate all RGCs continuously throughout the entire duration. Each cycle consisted of 4 s, comprising a 2 s “ON” period followed by a 2 s “OFF” period. The light source utilized in this study was a coupled LED (Thorlabs, United States) with a wavelength range spanning from 400 nm to 700 nm. The LED was operated at a minimum power output of 21.5 mW. In the case of ES, a bidirectional delayed pulse stimulation method was utilized. The stimulation parameters were set as follows: a pulse duration (PD) of 200 µs, an inter-pulse interval (IPI) of 500 µs, and a pulse amplitude of 300 mV. The stimulation duration was set to 1 min. During the stimulation process, the ground electrode of the ES was connected to the ground of the MEA interface circuit, while the stimulating electrode was connected to the intermediate region where a larger number of RGCs responded. CS involved the application of a high-K^+^ (20 µM) reagent. The reagent was directly added to the retinal culture medium using a pipette.

### 2.4 Fabrication of 3D MEA

**FIGURE 2:**
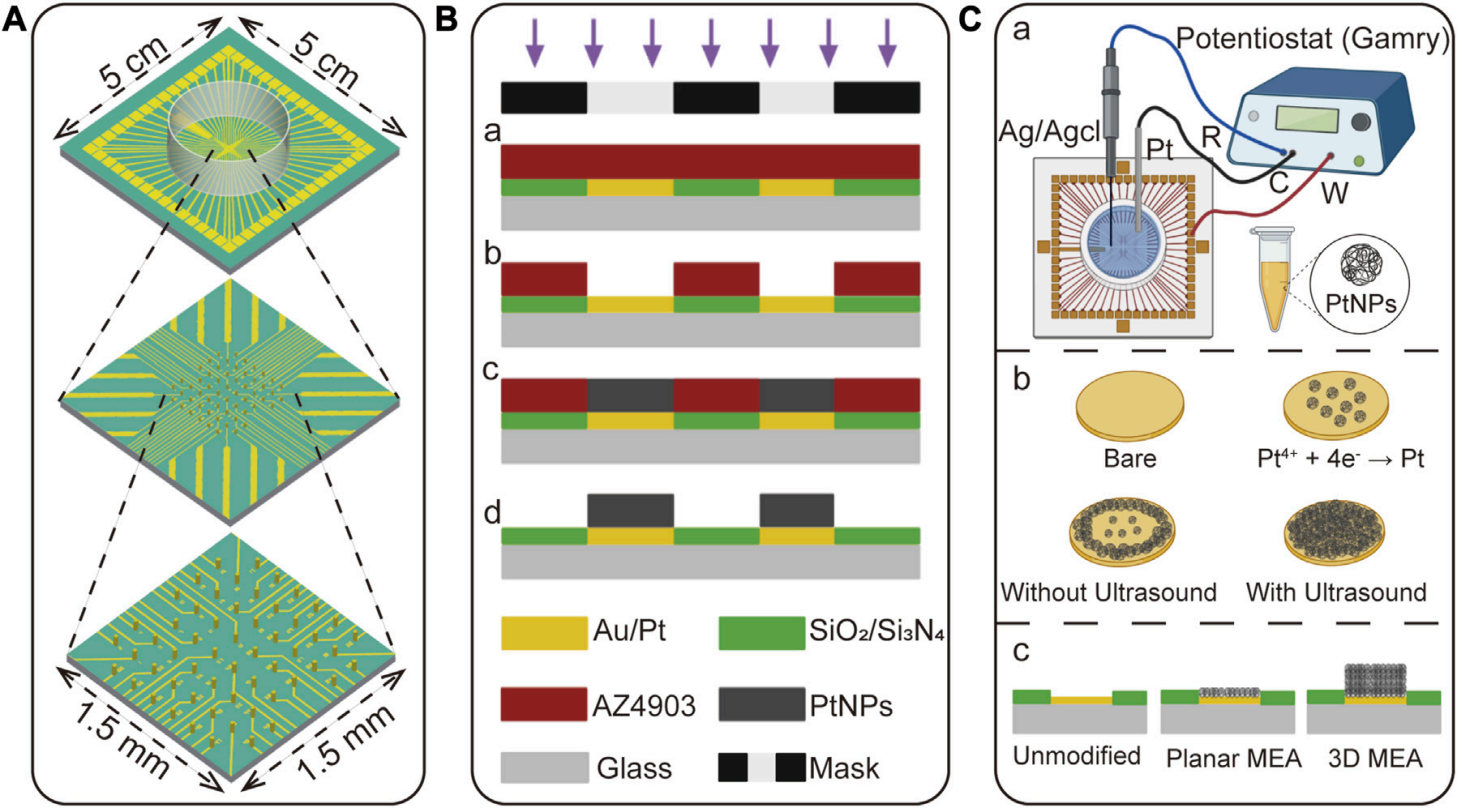
The design and fabrication of 3D MEA. **(A)** Illustration of the size and three-dimensional structure of the 3D MEA, comprising 57 working electrodes and one reference electrode. **(B)** Fabrication process of the 3D MEA. (a) Spin coating and photolithography on a planar MEA substrate. (b) Patterned photoresist through development to define the reactive area. (c) Electrodeposition of material to grow the three-dimensional structure. (d) Removal of the photoresist to release the three-dimensional structure. **(C)** Detailed description of the electroplating process to form a 3D structure. (a) Electroplating was performed using a three-electrode system, where the working electrode was the bare electrode to be plated, the counter electrode was Pt, and the reference electrode was Ag/AgCl. (b) In this study, PtNPs were chosen as the electroplating material, which was obtained by reducing Pt^4+^ onto the bare electrode surface. The addition of ultrasound during this process facilitated the uniform growth of PtNPs. (c) The unmodified electrode formed a groove after etching the insulating layer, and this groove was filled with a planar MEA. The 3D MEA, based on the planar MEA, was formed by this process to create a three-dimensional structure.

In this study, a 3D MEA was employed for recording, as illustrated in Figure 2A, which consisted of 57 working electrodes and one reference electrode. The electrode was fabricated on a 5 cm × 5 cm glass substrate through multiple steps of photolithography and electroplating, as depicted in Figure 2B. The fabrication process began with that of planar MEA according to the previous work (He et al., 2022), and then proceeded through steps a-d to form the three-dimensional electrodes. The detailed fabrication steps are as follows:

Firstly, the planar MEA underwent pre-treatment. Surface cleaning was performed using a plasma cleaner (100 W, 3 min) to enhance the hydrophilicity of the planar MEA, facilitating better adhesion of the photoresist. Subsequently, as shown in Figure 2B-a, the spin coating was carried out on the planar MEA to control the thickness of the photoresist by adjusting the spinning speed. After spin coating, pre-baking was conducted at 120°C for 3 min. Then, a photomask was used for selective exposure in the desired window regions. Different photoresists and thicknesses required corresponding adjustments in the exposure time to avoid overexposure or underexposure.

Next, the development process (Figure 2B-b) was performed using a 0.6% NaOH developer. The color change from dark red to golden yellow in the patterned areas indicated the completion of development, resulting in an MEA with multiple micropores. Subsequently, as illustrated in Figures 2B-c, C, three-dimensional microcolumns were grown in the micropores through electrodeposition, which was accomplished using an electrochemical workstation.

Finally, as illustrated in Figure 2B-d, the fully electroplated electrode was released by removing the photoresist using acetone, absolute ethanol, and deionized water, allowing the three-dimensional electrodes to be released. By following this process, the fabrication of the 3D MEA was completed.

### 2.5 Electroplating process for 3D MEA fabrication

As shown in Figure 2C-a, the electroplating process was carried out using a three-electrode system, where the working electrode was a bare electrode, the counter electrode was Pt, and the reference electrode was Ag/AgCl. The electroplating parameters, such as applied potential, current density, and deposition time, were carefully adjusted using an electrochemical workstation to control the deposition rate and the size of the formed nanoparticles. Figure 2C-b illustrates that the electroplating solution contained Pt^4+^, which was reduced to PtNPs and grown on the electrode surface during the electroplating process. Notably, the addition of ultrasound during the electroplating process enhanced the concentration and transfer rate of reactants on the electrode surface, resulting in a more uniform deposition of PtNPs (Tudela et al., 2014). Figure 2C-c illustrates the intended electrode structure with PtNPs deposited at different heights. The unmodified electrode exhibited a groove corresponding to the height of the insulating layer (The height of the insulating layer is approximately 300–500 nm), while the planar MEA was filled with PtNPs to eliminate this groove, but it did not achieve a true three-dimensional structure. Through the process shown in Figure 2B, the 3D MEA was successfully fabricated. (The height of the electrode is determined by the thickness of the spin-coating photoresist and the electroplating parameters).

### 2.6 Signal acquisition and analysis

RGC responses were recorded using a 3D MEA system, with the retina and electrodes positioned using the inverted microscope. A specialized retina anchor ensured stable placement. Continuous perfusion with a culture medium containing 5% CO_2_ and 95% O_2_ maintained cell viability. OS was applied to the retina’s photosensitive layer to elicit distinct RGC responses. RGC spikes were classified using Offline Sorter software and the K-means clustering algorithm, facilitating the identification of neuronal populations. NeuroExplorer (Nex Technologies, United States) software analyzed relevant metrics, while Origin (OriginLab, United States) and Python software visualized the data. Data were calculated as mean ± standard error of mean. Statistical comparisons utilized a two-tailed *t*-test for robust analysis. A statistical significance of *p* < 0.05 was set for all analyses.

## 3 Results

### 3.1 Morphological characterization of 3D MEA

**FIGURE 3:**
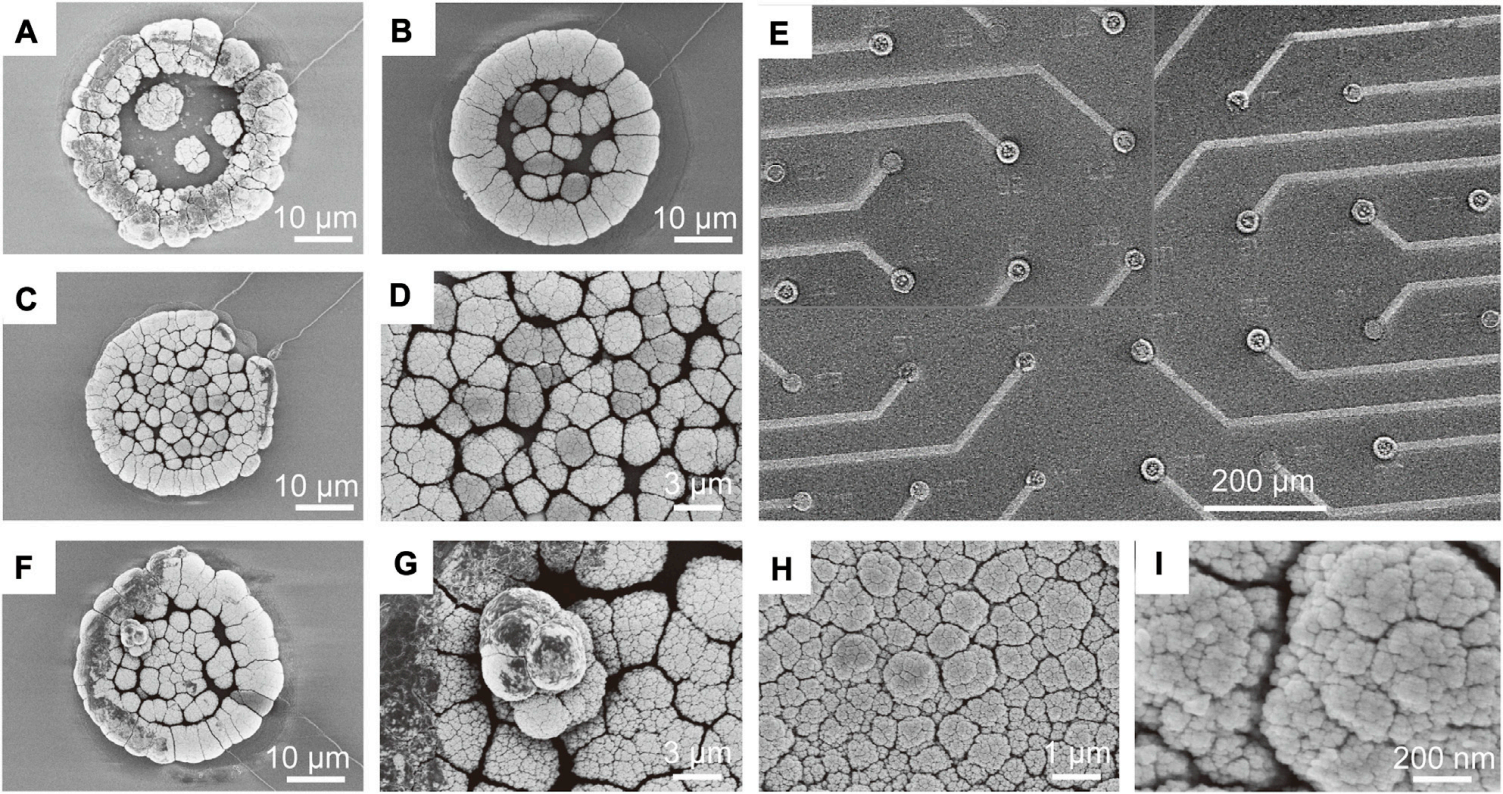
Morphological characterization of 3D MEA. **(A)** Morphology of PtNPs growing adherently to the wall to form a 3D ring structure. **(B)** Surface morphology of PtNPs deposition on the electrode without ultrasound. **(C)** Surface morphology of PtNPs deposition on the electrode with ultrasound. **(D)** SEM image of Figure 3C magnified to observe the formation of a more compact structure of PtNPs on the electrode with ultrasound. **(E)** SEM image of multiple electrode sites with protruding PtNPs formed by modification. **(F)** PtNPs forming a three-dimensional structure through layer-by-layer stacking. **(G)** Further magnification of the stacking details in Panel **(F)** reveals the clustering of multiple PtNPs, forming the three-dimensional structure. **(H)** Surface morphology of the electrode obtained with ultrasound in Panel **(G)**, showing a more uniform distribution of PtNPs. **(I)** Morphology at the nanoscale in Panel **(H)**, revealing a significant number of honeycomb-like structures.

**FIGURE 4:**
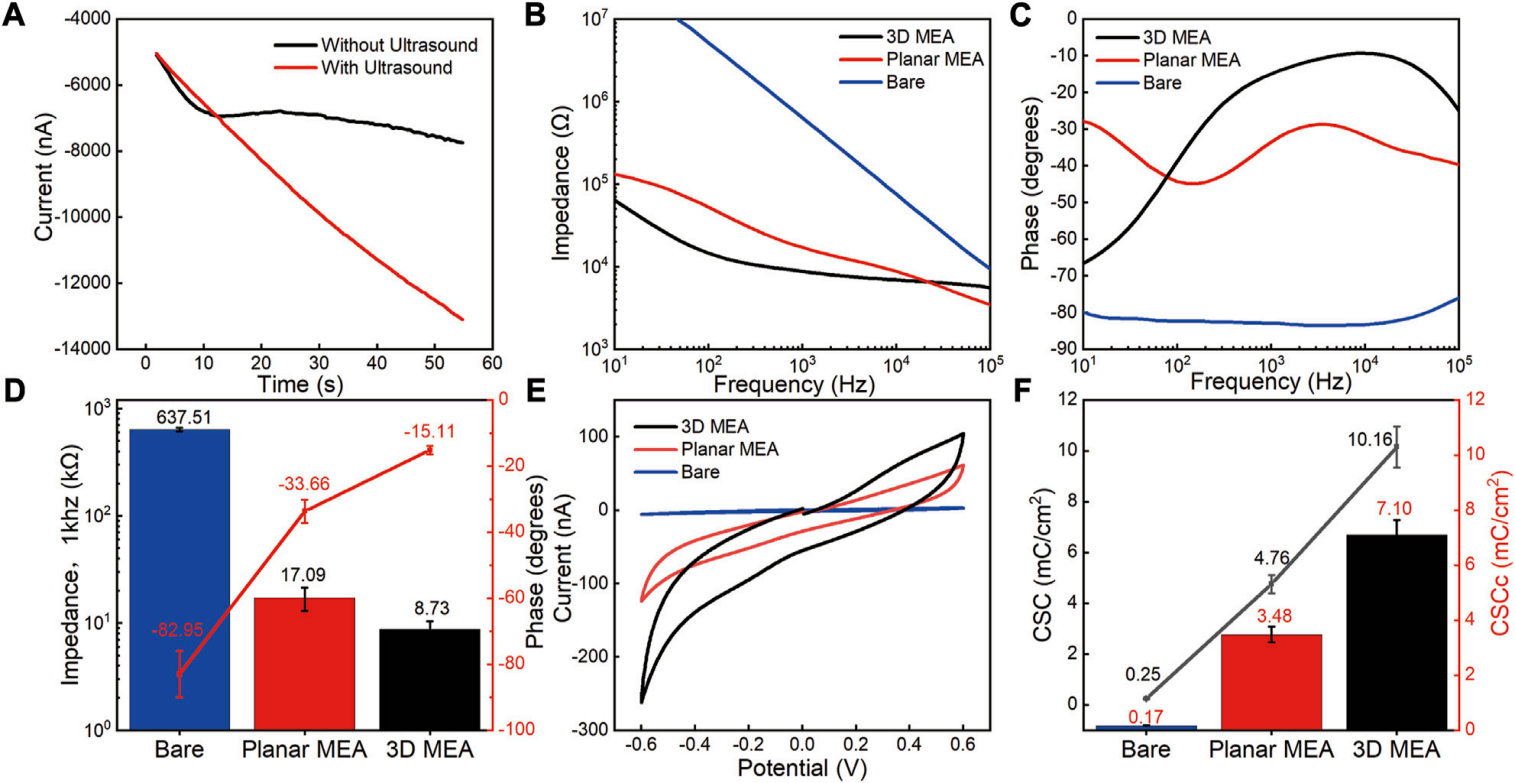
Electrochemical performance characterization of 3D MEA. **(A)** i-t response curves during the electrodeposition process with and without ultrasound conditions. **(B)** Impedance variation of bare electrodes, planar MEA, and 3D MEA in the frequency range of 10 Hz–100 kHz. **(C)** Phase variation of bare electrodes, planar MEA, and 3D MEA in the frequency range of 10 Hz–100 kHz. **(D)** Statistics of impedance and phase at 1 kHz frequency for bare electrodes, planar MEA, and 3D MEA (*n* = 3). **(E)** Cyclic voltammetry testing of bare electrodes, planar MEA, and 3D MEA. **(F)** Statistics of CSC and CSCc for bare electrodes, planar MEA, and 3D MEA (*n* = 3).

The morphology of the electrodes was characterized using scanning electron microscopy (SEM). Firstly, we characterized the morphology of PtNPs deposition on the electrode surface with and without ultrasound. Figures 3A–C show the 3D structural morphology of PtNPs formed from without ultrasound plating to the addition of ultrasound plating, and Figure 3D shows the surface morphology of the central region of the electrodes after ultrasound plating in more detail, clearly demonstrating that ultrasound-assisted electroplating leads to a more uniform and dense distribution of detection sites. Figure 4A illustrates the current-time curves of the electroplating process with and without ultrasound. Without ultrasound, the deposition rate starts to decrease after approximately 10 s. In contrast, under ultrasound conditions, the deposition rate remains constant, maintaining an enhanced current response curve. Subsequently, we investigated the modified morphology of multiple electrodes after PtNPs modification, as shown in Figure 3E, revealing multiple protruding electrode sites that facilitate the coupling between the electrode and RGCs.

Furthermore, we characterized the surface morphology of the three-dimensional structure formed by PtNPs on the electrode surface and its further ultrasound treatment. As depicted in Figures 3F, G, PtNPs formed a three-dimensional structure through layer-by-layer stacking, but the stacking exhibited non-uniformity. As mentioned earlier, the addition of ultrasound during the electroplating process improved this phenomenon. Figure 3H illustrates the dispersion of the initially deposited three-dimensional structure throughout the entire electrode surface under ultrasound, resulting in a more uniformly dense surface, as observed by SEM. This process was repeated cyclically, eventually forming PtNPs with a certain height. Figure 3I displays the nanoscale morphology of this structure, revealing a substantial number of dense and uniform honeycomb-like structures. This enhanced the specific surface area of the electrode, thereby contributing to improved electrochemical performance.

### 3.2 Electrochemical performance characterization of MEA

The electrochemical performance of the electrodes was characterized using electrochemical impedance spectroscopy (EIS) and cyclic voltammetry (CV). Figures 4B, C present the impedance phase test results for different types of electrodes. Compared to the bare electrode, both the 3D MEA and the planar MEA (modified with a thin platinum film) significantly improved the impedance and phase performance. At 1 kHz, the average impedance of the test points decreased from 637.51 ± 25.23 kΩ (bare electrode) to 17.09 ± 4.18 kΩ (planar MEA), and finally to 8.73 ± 1.66 kΩ (3D MEA). Simultaneously, the phase angle increased from −82.95° ± 7.06° (bare electrode) to −33.66° ± 3.53° (planar MEA), and finally to −15.11° ± 1.27° (3D MEA) (Figure 4D).

The CSC of the electrodes was evaluated through cyclic voltammetry (CV) testing, as shown in Figure 4E (Supplementary Text S1 presents a methodology for calculating the CSC and CSCc, along with a technique for determining the active area of the electrode). The calculated CSC increased from 0.25 ± 0.02 mC/cm^2^ (bare electrode) to 4.76 ± 0.36 mC/cm^2^ (planar MEA), and finally to 10.16 ± 0.81 mC/cm^2^ (3D MEA). The CSCc has become a popular method for determining the charging capacity of stimulating microelectrodes. The calculated CSCc increased from 0.17 ± 0.01 mC/cm^2^ (bare electrode) to 3.48 ± 0.28 mC/cm^2^ (planar MEA), and finally to 7.10 ± 0.55 mC/cm^2^ (3D MEA) (Figure 4F). The differences observed in impedance phase, CSC, and CSCc between the bare electrode, planar MEA, and 3D MEA highlight the advantages of the 3D protruding structure in improving electrode performance.

### 3.3 Evaluating the detection performance of 3D MEA

**FIGURE 5:**
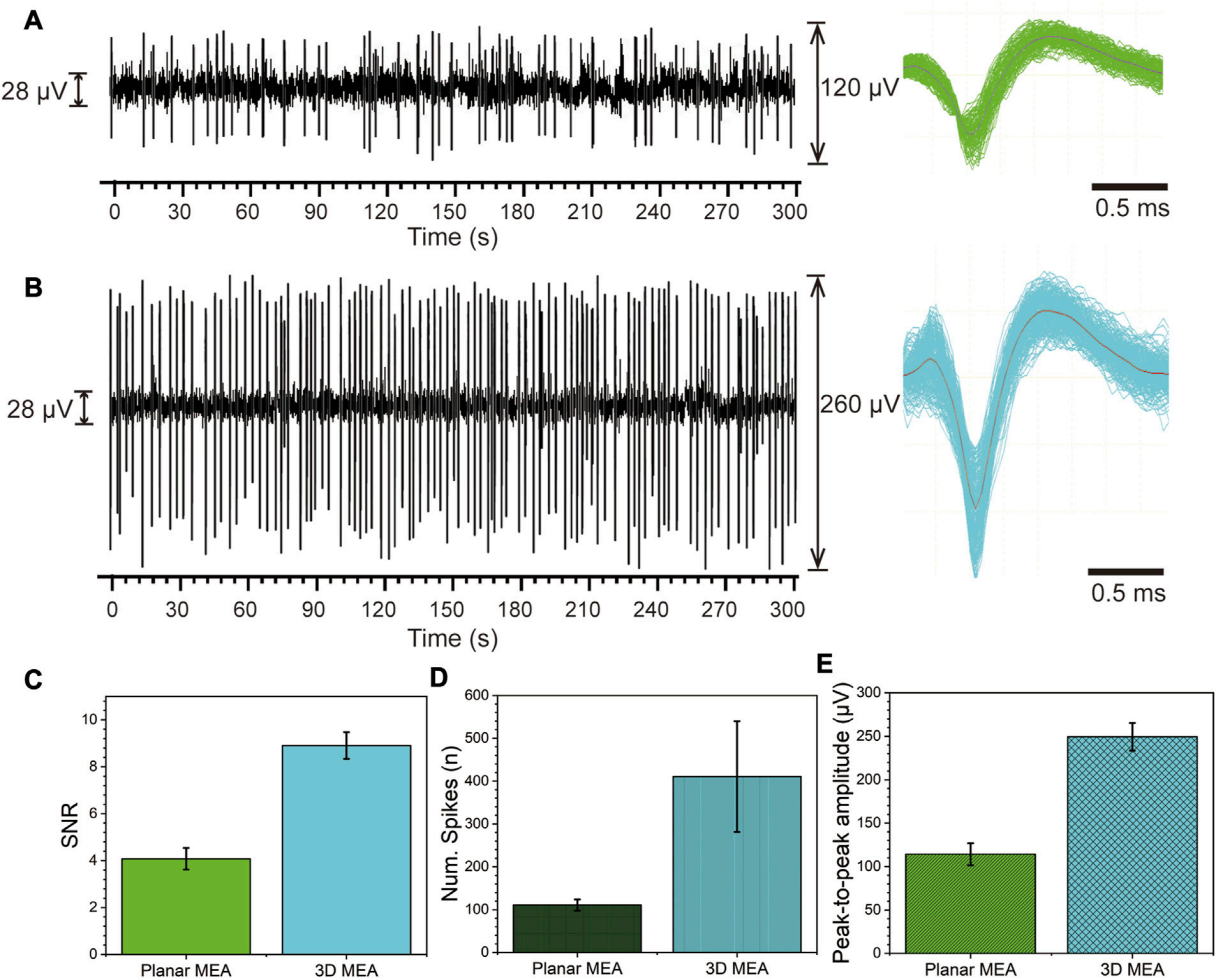
Spontaneous firing activity of RGCs detected by the planar MEA and 3D MEA. **(A)** Spontaneous firing activity of RGCs detected by planar MEA. Left: Spike firing within 300 s. Right: Stacked waveform of spikes. **(B)** Spontaneous firing activity of RGCs detected by 3D MEA. Left: Spike firing within 300 s. Right: Stacked waveform of spikes. **(C)** Statistical analysis of the signal-to-noise ratio (SNR) between planar MEA and 3D MEA. **(D)** Statistical analysis of the average number of detected spikes within 5 min between planar MEA and 3D MEA. **(E)** Statistical analysis of the average peak-to-peak amplitude of detected spikes between planar MEA and 3D MEA (*n* = 5).

We conducted performance tests on planar MEA and 3D MEA in electrophysiological detection experiments of RGCs. As shown in, Figure 5A represents the test results of one channel of the planar MEA, with the left side showing the spike trajectories within 300 s and the right side showing the overlay of spikes after noise removal throughout the entire time. The corresponding test results of one channel of the 3D MEA are shown in Figure 5B. Compared to Figure 5A, the spikes in Figure 5B exhibit significantly increased amplitude and count.

We performed statistical analysis on the data obtained from five channels of both planar MEA and 3D MEA. Firstly, the statistical results of the SNR are shown in Figure 5C, where the SNR of the planar MEA is 4.08 ± 0.46 and that of the 3D MEA is 8.91 ± 0.57. Next, we analyzed the average number of detected spikes within 5 min. The planar MEA detected 110.60 ± 3.11 spikes, while the 3D MEA detected 410.54 ± 29.09 spikes (Figure 5D). Finally, we examined the average peak-to-peak amplitude of the spikes. The planar MEA had an amplitude of 114.20 ± 12.84 μV, while the 3D MEA had an amplitude of 249.40 ± 15.99 μV (Figure 5E). These results indicate that the detection performance of the 3D MEA surpasses that of the planar MEA. The increased height not only enhances the surface area, thereby improving electrode performance but also promotes tighter adhesion to retinal tissue, leading to improved coupling and stability (Supplementary Figure S2) (Hai et al., 2010; Santoro et al., 2013). This further emphasizes the beneficial role of the three-dimensional electrode structure in the cultivation and detection of *ex vivo* retinal tissue.

### 3.4 Response patterns and times of RGCs under multi-modal stimulations

**FIGURE 6:**
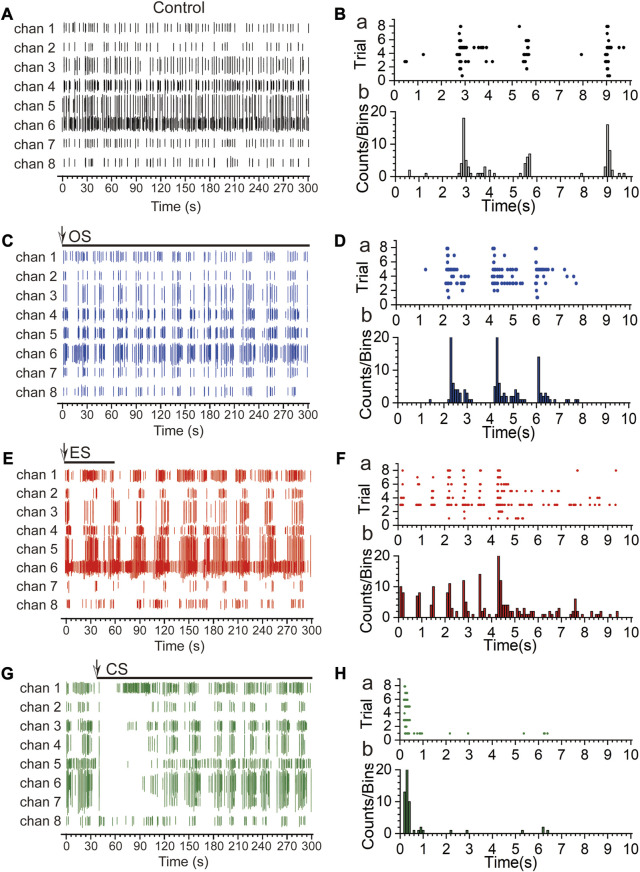
Response patterns and times of RGCs under multi-modal stimulations. **(A)** Spontaneous firing activity of multiple channels in the control group within 300 s. **(B)** Spike raster plot (a) and time histogram (b) within 10 s for the control group. **(C)** Firing activity of multiple channels under optical stimulation within 300 s [The arrows indicate the start time of the stimulation, and the horizontal lines below the arrows represent the duration of the stimulation. The same applies to subsequent cases. The optical stimulation is continuous throughout the entire 5 min. Light source is coupled LED, wavelength range: 400 nm–700 nm, power: 21.5 mW (Min)]. **(D)** Spike raster plot (a) and time histogram (b) within 10 s under optical stimulation. **(E)** Firing activity of multiple channels under electrical stimulation within 300 s (The parameters for electrical stimulation were referenced from the Methods section, with a stimulation duration of 1 min). **(F)** Spike raster plot (a) and time histogram (b) within 10 s under electrical stimulation. **(G)** Firing activity of multiple channels under chemical stimulation within 300 s [high-K^+^ (20 µM)]. **(H)** Spike raster plot (a) and time histogram (b) within 10 s under chemical stimulation.

To investigate the response of RGCs to different types of stimuli, we sequentially performed OS, ES, and CS on the retina, with each stimulation recorded for 5 min and a 5-min interval between stimulations. Figures 6A, C, E, G represent the firing patterns of action potentials within 5 min for the control group (A), OS (C), ES (E), and CS (G), respectively. The overall responses of RGCs varied across the four conditions. In the control group, spontaneous firing was random, except for channel 6, where the firing activity was relatively sparse compared to the other channels. In the case of OS, the most significant difference from the control group was the emergence of rhythmic firing patterns in RGCs after stimulation. The neurons exhibited oscillatory firing within a certain period, which has been demonstrated to encode specific information. A detailed analysis of this phenomenon is presented in the next section. Next, ES was applied, and similar to OS, rhythmic firing patterns were observed. However, in this case, the number of spikes during each period of rhythmic firing increased, and the period of the rhythm became longer. This effect was particularly noticeable in certain channels, such as channels 1, 5, and 6. Finally, CS was performed by adding KCl to the culture medium. We observed that RGCs did not respond immediately after the stimulation for approximately 45 s. Studies have shown that neurons require a certain level of adaptation to CS (Montes et al., 2019), and they need time to reestablish stable firing activity after the stimulus onset. This adaptive and recovery process may require a specific time window, and as a result, no firing activity may be observed for a while following the stimulation. We speculate that this period represents the adaptation time of RGCs.

To further analyze the differences in response times of RGCs under the three types of stimuli, we analyzed the raster plots and time histograms of the first 10 s following each stimulation [Figure 6B, D, F, H, control group (B), OS (D), ES (F), and CS (H)]. In the case of spontaneous firing, the discharges were concentrated around 3 s and 9 s, with fewer channels exhibiting firing around 6 s. The overall firing activity lacked periodicity. For OS, firing was observed around 2 s, 4 s, and 6 s. The period with the highest number of spikes was approximately 2 s, which corresponds to half the period of the OS. This finding suggests that the response of RGCs to OS is associated with the “ON” and “OFF” states, representing the response of two types of RGCs. Analysis of the 10 s response to ES revealed that RGCs exhibited almost simultaneous responses immediately after the stimulation onset. The response time for ES was the fastest among the different stimuli. Additionally, within the first 5 s, there was an increase in the firing rate, indicating that ES evoked responses from a larger number of RGCs. However, unlike the rhythmic firing observed during the 10 s period of OS, no periodic firing was observed during the 10 s period of ES. Considering the entire 300 s duration, the response period of ES was longer. Finally, for CS, it was evident that RGCs did not respond until approximately 1 s after the stimulation onset (The time of addition of high-K^+^ is the time of stimulus onset), and this is due to the fact that it takes time for the diffusion of high-K^+^ to take place, and that when acted upon the neuron, the neuron responds by silencing in order to adapt to this stimulus. These results demonstrate that RGCs exhibit different response patterns to different stimuli, including variations in periodicity, firing rate, and response time. The specific encoding of information will be further analyzed in the next section.

### 3.5 Population encoding of RGCs under multi-modal stimulations

**FIGURE 7:**
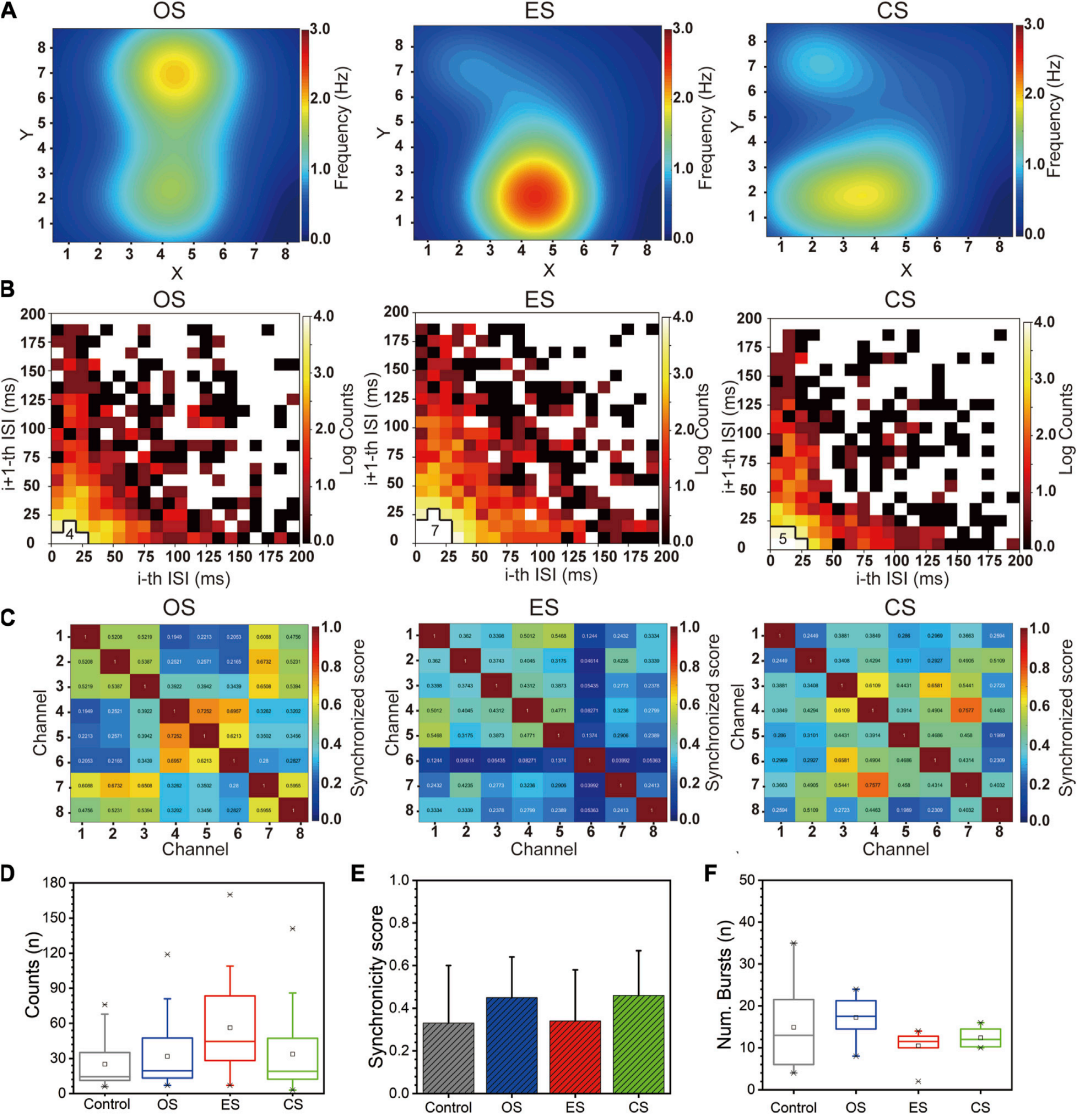
Population encoding of RGCs under multi-modal stimulations. **(A)** Heatmap of RGCs firing rates under different stimulation (From left to right: OS, ES, CS). **(B)** Joint ISI distributions of RGCs under different stimulation (From left to right: OS, ES, CS). **(C)** Correlation heatmap matrix of RGCs under different stimulation (From left to right: OS, ES, CS). **(D)** Boxplot of the number of neural firings within 40 ms in the joint ISI distribution. **(E)** Statistical analysis of correlation coefficients. **(F)** Boxplot of the number of bursts (*n* = 8).

We first analyzed the rate encoding of RGCs under different conditions. Supplementary Figure S3A (Control group) and Figure 7A (From left to right: OS, ES, CS) depict the spike firing rate heatmap of RGCs. Overall, all three stimuli increased the response of RGCs. Among them, ES resulted in the highest firing rate of 1.67 ± 0.74, followed by OS with a firing rate of 1.12 ± 0.34, and CS with a firing rate of 1.11 ± 0.28. The control group exhibited a firing rate of 0.94 ± 0.33 spikes/s (Supplementary Figure S3D). Examining the active regions, OS enhanced the response of RGCs across the entire visual field, as all RGCs showed a response. This observation can be attributed to the fact that OS covered the entire visual field. However, for ES, RGCs near the stimulation site exhibited higher responses, significantly higher than those in other areas. This suggests that not all RGCs were stimulated by ES. In the case of CS following ES, it can be considered as a global stimulation of RGCs, and therefore, the firing locations continued from the previous firing locations.

Next, we analyzed the temporal encoding of RGCs under different conditions. We first examined the joint ISI distribution of RGCs. Supplementary Text S2 provides algorithm details for joint ISI distribution. Supplementary Figure S3B (Control group) and Figure 7B (From left to right: OS, ES, CS) displays the joint ISI distribution for all channels, with the number of grid cells with log counts greater than 4 near the origin being counted to visually represent the differences among stimuli. A higher value indicates smaller ISI and greater neuronal excitation. Therefore, compared to the control group, RGCs exhibited increased excitability under all stimulus conditions, with ES inducing higher excitability than CS, which was higher than OS. Figure 7D presents the count of neuronal firings within all 40 ms intervals in the joint ISI distribution. The results show that ES elicited 56.25 ± 10.38 spikes firings, CS elicited 33.69 ± 8.94 spikes firings, OS elicited 31.81 ± 7.76 spikes firings, and the control group exhibited 25.13 ± 5.38 spikes firings. This indicates that under equivalent conditions, ES triggered a higher number of RGC firings. Moreover, these statistical results align with the trend observed in firing rates, demonstrating the consistency between overall firing quantity and excitability.

Then, we analyzed the correlation between RGCs under different conditions. We calculated the Pearson correlation coefficient to measure the correlation between neurons and generated a correlation matrix heatmap, as shown in Supplementary Figure S3C (Control group) and Figure 7C (From left to right: OS, ES, CS) (refer to Supplementary Text S3 for the detailed algorithm). We observed that the correlation coefficients changed most noticeably in OS and CS, followed by ES and the control group. The statistical results are presented in Figure 7E, illustrating the impact of different stimulus modalities on the correlation between neurons. OS and CS induced higher correlation, possibly due to their global stimulation nature, which triggered more consistent RGC response patterns. On the other hand, ES only acted on specific regions, leading to increased heterogeneity in RGC activity and reduced correlation levels.

Finally, we analyzed the number of bursts under the four conditions. Supplementary Text S4 describes a description of the burst counting algorithm. Figure 7F presents the overall burst statistics. OS had the highest number of bursts, with a count of 17.25 ± 1.74. The control group exhibited 14.88 ± 3.64 bursts, CS had 12.38 ± 0.78 bursts, and ES had 10.50 ± 1.31 bursts. The lower number of bursts in CS was attributed to a silent period of RGCs, while the lower count in ES was due to an excessively long firing period. In summary, RGCs exhibited distinct differences in Rate encoding and temporal encoding in response to different sensory stimuli. These encoding mechanisms provided insights into the discrimination of various input stimuli.

## 4 Conclusion

In summary, we employed a simple and scalable microfabrication method to design and fabricate a 3D MEA for the electrophysiological detection of RGCs. The method utilized a planar MEA as a substrate and employed processes such as photolithography and electroplating to grow materials on the electrode surface, thus forming a three-dimensional structure. The selection of the appropriate electroplating material was crucial for achieving excellent electrode performance. The chosen material is needed to possess good conductivity, solubility, and stability. Additionally, considering that our detection target was *ex vivo* retinal tissue, which could potentially damage the electrode structure during positioning, it was preferable for the material to also have a certain mechanical strength.

In this study, PtNPs were chosen as the selected material. However, it is worth noting that further research can explore other superior electrode materials to meet different testing requirements. Moreover, the substrate used in this method can also be adjusted according to the specific testing needs, such as silicon substrates or plastic substrates, which are equally applicable. Following electrochemical performance testing of bare electrodes, planar MEA, and 3D MEA, comparative analysis revealed that the 3D MEA exhibited superior performance in all aspects. Furthermore, in the electrophysiological testing experiments with *ex vivo* retinal tissue, the detection performance of the 3D MEA was significantly superior to that of the planar MEA. Therefore, the fabrication method employed in this study for three-dimensional electrodes offers strong customization capabilities and cost-effectiveness.

Benefiting from the excellent performance of the electrode, this study also conducted encoding research on RGCs under multi-modal stimulation. Specifically, we employed OS, ES, and CS as sensory inputs to the retina to investigate the response patterns and response times of RGCs under different inputs. We observed distinct response patterns and response times for each type of stimulation, such as the notable silent period observed during CS. These findings contribute to our understanding of the regulatory function and adaptation mechanisms of RGCs. Furthermore, we analyzed the Rate encoding and temporal encoding of RGCs under different sensory inputs. Regarding Rate encoding, we found that ES was more effective than the other two stimuli in eliciting RGC firing. This is promising news for research aiming to restore vision through the ES of RGCs. In terms of temporal encoding, by analyzing the joint ISI distribution, synchrony, and burst counts of RGCs, we found that these analyses jointly encode different sensory inputs. These analyses interact but are not substitutes for each other; for example, we found that neuronal firing rates and excitability (joint ISI) were higher with ES, but synchronicity and burst counts were relatively low. It is the combined assessment of multiple methods of analysis that can more accurately encode stimuli with different sensory inputs. In conclusion, both our electrode fabrication method and the encoding research on RGCs under multi-modal stimulation are expected to invigorate the field of neural encoding.

## Data Availability

The raw data supporting the conclusion of this article will be made available by the authors, without undue reservation.
