# Supplementary material for: Exploring retinal ganglion cells encoding to multi-modal stimulation using 3D microelectrodes arrays
Source: Front Bioeng Biotechnol. 2023 Aug 1;11:1245082. doi: 10.3389/fbioe.2023.1245082 (PMC10434521; doi:10.3389/fbioe.2023.1245082)
Supplement: Supplementary file 1 [file DataSheet1.pdf]

Supplementary Material

Exploring Retinal Ganglion Cells Encoding to Multi-Modal Stimulation Using 3D Microelectrodes Arrays

Kui Zhang, Yaoyao Liu, Yilin Song, Shihong Xu, Yan Yang, Longhui Jiang, Shutong Sun, Jinping Luo*, Yirong Wu*, Xinxia Cai*

*** Correspondence:** Xinxia Cai: xxcai@mail.ie.ac.cn Yirong Wu: wyr@mail.ie.ac.cn Jinping Luo: jpluo@mail.ie.ac.cn

1 Supplementary Figures and texts

1.1 Supplementary Figures

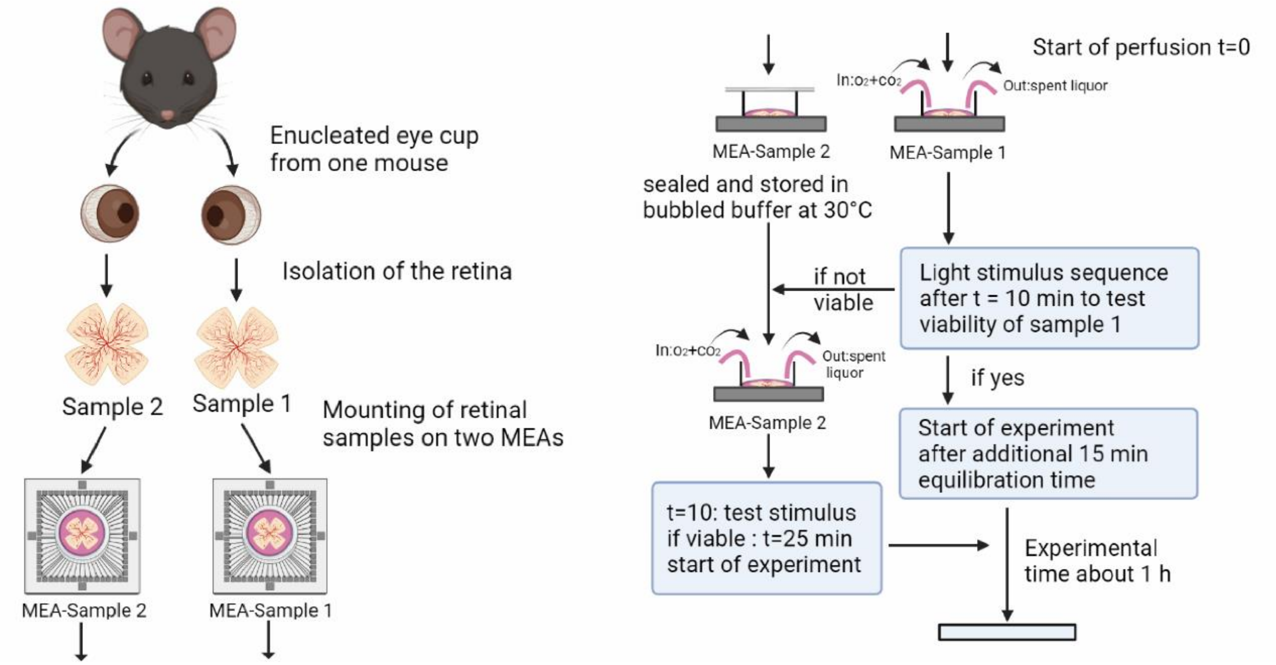

Figure S1. Adhesion and Viability Testing of Ex Vivo Retina Cultured on MEA

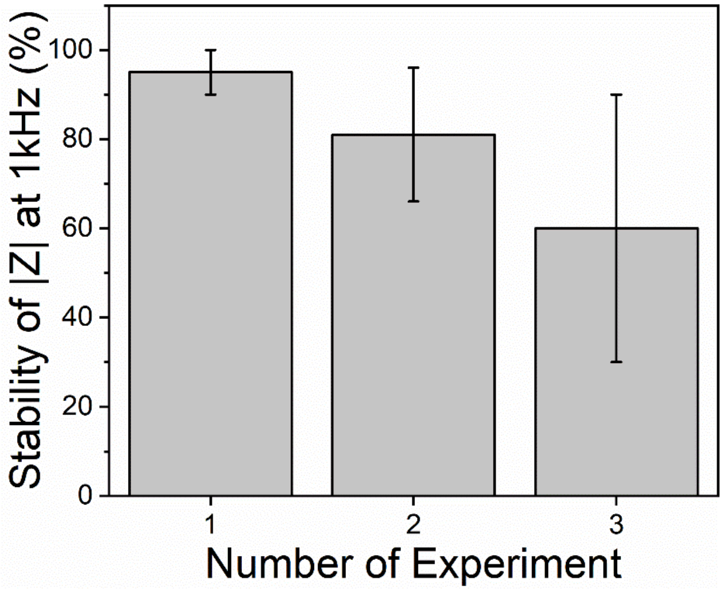

Figure S2. Stability Characteristics of 3D MEA. To evaluate the stability of the electrodes, we conducted impedance measurements to assess the invariance of the electrode impedance after completing retinal experiments. Specifically, we calculated the invariance of impedance as the ratio of the current impedance to the pre-experiment impedance, multiplied by 100%. We found that the majority of electrodes exhibited an impedance invariance of over 90% after one retinal experiment, indicating good stability and reusability. However, as the number of experiments increased, the amount of change in electrode impedance gradually became larger. Therefore, when the impedance change exceeds 50%, we recommend reworking the electrodes according to the process described in our paper to ensure optimal performance for reuse.

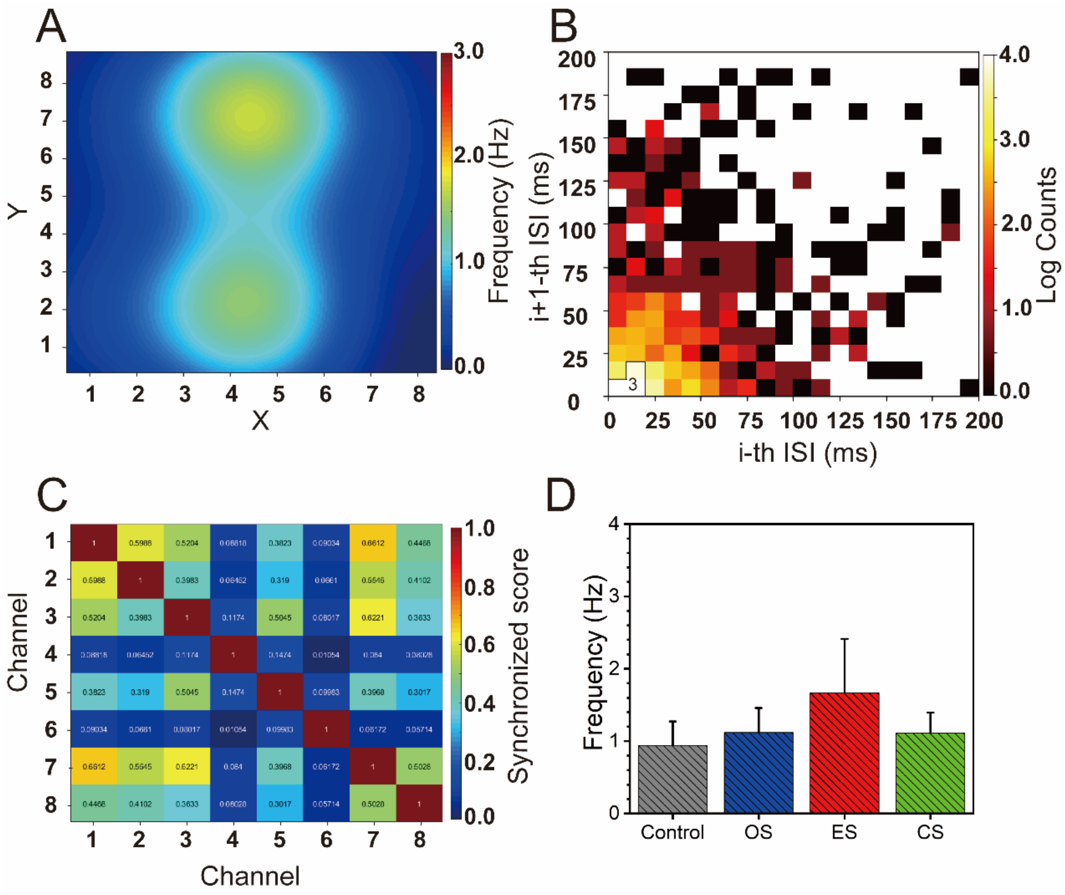

Figure S3. Population coding of RGCs in the control group. (A) Heatmap of RGCs firing rates. (B) Joint ISI distributions of RGCs. (C) Correlation heatmap matrix of RGCs. (D) Statistical analysis of average firing rates (n=8).

1.2 Supplementary Texts

Supplementary Text 1. Calculation of CSC and CSC_C and determination of electrode area

The CSC and CSC_C of the microelectrode were measured in PBS solution using cyclic voltammetry (CV). The measurement employed a three-electrode system with a Pt electrode as the counter electrode and an Ag|AgCl electrode as the reference electrode. The CV scan range was -0.6V to -0.8V, and the scan rate was 50 mV/s in a slow scan mode. To calculate the CSC, the integral of the area enclosed by the current and voltage in the CV curve was divided by the microelectrode area. For the calculation of CSC_C, only the area of the cathode current was integrated and divided by the microelectrode area.

In the aforementioned electrochemical system, the electrode surface area was determined using the chronoamperometry technique, which involves applying a step potential to the electrode and recording the variation of charge with time. The determination of the electrode surface area was based on the Cottrell equation, as mentioned in the literature *(Fragkou et al., 2012;Baccarin et al., 2018)*. The Cottrell equation relates the measured charge (Q) to the square root of time ($t^{1/2}$) and is given by:

$$Q = 2nFACD^{1/2}t^{1/2}\pi^{-1/2}$$

Here, n represents the number of transferred electrons, F is the Faraday constant (96485 C eq^{-1}), A is the electrode surface area (cm^2), C is the concentration of the redox species (mol cm^{-3}), and D is the diffusion coefficient. By fitting the experimental data of charge (Q) versus $t^{1/2}$ to the Cottrell equation, the slope of the curve was determined. Taking the reciprocal of the slope yields the reciprocal of the electrode surface area, which allows us to calculate the electrode surface area accurately.

Supplementary Text 2. Algorithm Description for Joint ISI Distribution

1. Create a matrix:

   Create a matrix, binCounts, of size (X, Y), where each element is initialized to zero. This matrix will be used to store the counts of different time interval combinations.

2. For each spike occurring at time t[i]:

a) Calculate the interval, Interval_I: Compute the time interval between the current spike time, t[i], and the previous spike time, t[i-1], i.e., Interval_I = t[i] - t[i-1].

b) Calculate binX: Normalize Interval_I to an appropriate range by subtracting the minimum interval time, MinInterval, and dividing by the bin size, Bin, i.e., binX = (Interval_I - MinInterval) / Bin.

3. For each spike occurring at time t[i+1]:

a) Calculate Interval_I_Plus_1: Compute the time interval between the current spike time, t[i+1], and the previous spike time, t[i], i.e., Interval_I_Plus_1 = t[i+1] - t[i].

b) Calculate binY: Normalize Interval_I_Plus_1 to an appropriate range by subtracting the minimum interval time, MinInterval, and dividing by the bin size, Bin, i.e., binY = (Interval_I_Plus_1 - MinInterval) / Bin.

4. Increase the corresponding bin count:

   Increment the value of the binCounts matrix at coordinates (binX, binY) by 1, i.e., binCounts[binX, binY] = binCounts[binX, binY] + 1.

5. Plot the graph:

   Visualize the values in the binCounts matrix on a graph using a color scale. Each matrix element's value determines the color displayed at the corresponding position. Different colors can be used to represent different count values.

By executing this algorithm, statistical analysis of the time intervals between neural spike events can be performed, and the results can be visualized as a Joint ISI Distribution graph, which displays the frequency or count of different time interval combinations.

The parameters used in this study are as follows:

Min int. (sec) = 0

Max int. (sec) = 0.2

Bin (sec) = 0.01

Bins per decade = 20

Matrix Scale = Color

Supplementary Text 3: Algorithm for Correlation Analysis

To analyze the correlation between neurons, we employed the Pearson correlation coefficient as a measure of their correlation. The Pearson correlation coefficient (r) is calculated using the following formula:

Here,X_i and Y_i represent the firing activities of the two neurons at different time points, and μX and

$$r = \frac{\sum(X_i - uX)(Y_i - uY)}{\sqrt{\sum(X_i - uX)^2}\sqrt{\sum(Y_i - (uY)^2}}$$

μY denote the corresponding means of the time series.

The calculation of the Pearson correlation coefficient involves the following steps:

a) Data Preparation: We collected time series data representing the firing activities of two neurons at different time points.

b) Mean Computation: For each neuron's time series, we calculated the mean values μX and μY.

c) Standard Deviation Computation: We computed the standard deviations σX and σY of the time series.

d) Standardization: Each time series was standardized by subtracting the mean and dividing by the standard deviation, resulting in normalized time series.

e) Covariance Computation: We calculated the covariance cov(X, Y) between the standardized time series to measure their directional association.

f) Pearson Correlation Coefficient Calculation: The Pearson correlation coefficient r was computed by dividing the covariance cov(X, Y) by the product of the standard deviations σX and σY.

The resulting Pearson correlation coefficient r ranges between -1 and 1. A value close to 1 indicates a positive correlation, suggesting a linear relationship between the firing activities of the two neurons. A value close to -1 indicates a negative correlation, implying an inverse linear relationship. A value close to 0 indicates no linear correlation between the firing activities of the neurons.

To visually represent the correlation patterns between neurons, we constructed a correlation heatmap. The heatmap displays the correlation coefficients as color-coded values, providing an intuitive visualization of the synchronization and interactions among neurons.

In this study, we employed the aforementioned method to analyze the correlation between neurons. By calculating the Pearson correlation coefficient, we quantified the degree of correlation between neurons and represented it through a correlation heatmap. This approach offers valuable insights into the synchronization and interactions of neurons in understanding the functionality and dynamics of neural networks.

Supplementary Text 4: Algorithm for Burst Statistics

We employed the MaxInterval method to perform burst detection. The algorithm for this method is as follows:

a) Start scanning the spike train and continue until an interspike interval is encountered that is less than or equal to the specified **Max Interval**.

b) As long as the interspike intervals remain less than or equal to the Max End Interval, include those spikes in the current burst.

c) If an interspike interval exceeds the Max End Interval, it indicates the end of the current burst.

d) Merge any bursts that are separated by intervals smaller than the specified Min Interval Between Bursts. This step helps to combine closely spaced bursts into a single burst.

e) Eliminate any bursts that have a duration shorter than the specified Min Duration of Burst or contain fewer spikes than the specified Min Number of Spikes. This step ensures that only bursts meeting the defined criteria are considered for analysis.

The parameters used in this study are as follows:

Max Int. (sec) = 0.5

Max End Int. = 3

Min Interburst Int. = 1

Min Burst Duration = 0.1

Min Num. Spikes = 3

Baccarin, M., Cervini, P., and Cavalheiro, E.T.G. (2018). Comparative performances of a bare graphite-polyurethane composite electrode unmodified and modified with graphene and

carbon nanotubes in the electrochemical determination of escitalopram. *Talanta* 178**,** 1024-1032.

Fragkou, V., Ge, Y., Steiner, G., Freeman, D., Bartetzko, N., and Turner, A.P.F. (2012). Determination of the Real Surface Area of a Screen-Printed Electrode by Chronocoulometry. *International Journal of Electrochemical Science* 7**,** 6214-6220.
